# Effects of aqueous and hydro-alcoholic extracts of *Seidlitzia rosmarinus* on male mouse reproductive system: An experimental study

**DOI:** 10.5935/1518-0557.20220028

**Published:** 2023

**Authors:** Ali Akbar Oroojan, Mojtaba Dolatshahi, Mohammad Amin Behmanesh, Narges Chenani, Marzieh An’aam

**Affiliations:** 1 Department of Physiology, Faculty of Medicine, Student Research Committee, Dezful University of Medical Sciences, Dezful, Iran; 2 Department of Physiology, Faculty of Medicine, Dezful University of Medical Sciences, Dezful, Iran; 3 Department of Histology, Faculty of Medicine, Dezful University of Medical Sciences, Dezful, Iran; 4 Student Research Committee, Faculty School of Paramedical Sciences, Dezful University of Medical Sciences, Dezful, Iran; 5 Faculty of Medicine, Dezful University of Medical Sciences, Dezful, Iran

**Keywords:** *Seidlitzia rosmarinus* antioxidants, spermatozoa, male reproductive system, mice

## Abstract

**Objective:**

This study investigated the effects of aqueous and hydro-alcoholic extracts of *Seidlitzia rosmarinus* on reproductive hormones, sperm variables, and antioxidant enzymes level in the mice testis.

**Methods:**

In this experimental study, 24 three-month-old male NMRI mice weighing (25-30g) were divided into three groups: control, aqueous and hydro-alcoholic extracts of *Seidlitzia rosmarinus* 100mg/kg. Dissolved extracts were gavaged orally for 35 days. One day after receiving the last dose of the extract, the blood sample, testis, and the epididymis tail were taken for plasma hormonal, testicular antioxidants level, sperm count, and vitality assessments.

**Results:**

Testicular level of malondialdehyde increased in aqueous and hydro-alcoholic extracts groups (*p*=0.04); total antioxidant capacity decreased in aqueous and hydro-alcoholic extracts groups (*p*=0.008); and the consumption of aqueous (*p*<0.001) and hydro-alcoholic (*p*=0.03) extracts decreased catalase in comparison with the control group. The plasma level of luteinizing hormone decreased in the aqueous extracts administrated group (*p*=0.009); the follicle-stimulating hormone increased in aqueous (*p*=0.03), and hydro-alcoholic extracts administered mice; and the testosterone level decreased in aqueous extract-treated animals versus the control group (*p*<0.001). The sperm count was increased in aqueous (*p*=0.04) and hydro-alcoholic (*p*=0.009) extracts groups, but its vitality was decreased (*p*=0.008) in comparison with the control group.

**Conclusions:**

In conclusion, *Seidlitzia rosmarinus* has an adverse effect on male reproductive hormones and sperm viability via increased lipid peroxidation and reduced antioxidant defense system performance.

## INTRODUCTION

The rapid and uncontrolled increase of the world’s population and its problems is one of the fundamental problems of the present century. Also, the fact that contraception is only the responsibility of women has changed and men have also been considered in the discussion of population reduction. Due to the adverse effects of drugs in recent decades, traditional medicine, especially herbal medicine reduces the rates of pregnancy. Therefore, ideally, everyone wants to have a drug that is non-toxic and inhibits sperm production without side effects (Bala *et al.*, 2014; Ojo *et al.*, 2020). *Seidlitzia rosmarinus* is a perennial woody plant of the Chenopodiaceae family that includes 102 genera and 1400 species. Most of this family has a high salt tolerance and is widely found in the Middle East and Central Asia and is one of the most famous plants in saline soils. This plant has played a role in food and industrial use. The ash from burning leaves and stems has antiseptic and antibacterial properties, and it is also used to treat acne (Zolfaghari *et al.*, 2018). The previous study findings show that *Seidlitzia rosmarinus* extract significantly and concentration-dependently reduces the viability and inhibition of cell proliferation of human cervix carcinoma and human hepatocellular carcinoma cells. Also, the saponins in the extracts of this plant cause cytotoxic effects, which is also confirmed in the case of *Seidlitzia rosmarinus* extract (Zolfaghari *et al.*, 2018).

Oxidative stress has an adverse effect on male reproductive functions and causes infertility by affecting the hypothalamus-pituitary-gonadal axis and sperm dysfunction. One of the main mechanisms for this condition is inducing an imbalance in the antioxidant defense system (Agarwal *et al.*, 2014; Ahangarpour *et al.*, 2018; Darbandi *et al.*, 2018). Reactive oxygen species induce lipid peroxidation by increasing the testicular level of malondialdehyde (MDA), that leads to membrane characteristics disruption and destroys sperm function such as fertility power. Also, enhancing the level of this variable produces DNA damages in the sperm nucleus, that this alteration increases the rate of male infertility (Aitken, 2017). The modulation of this system lets the tumor cells bypass cell death through excessive production of reactive oxygen species. So, excessive reactive oxygen species production results in induced cytotoxic effects, cancer cell cycle arrest, and apoptosis (Carrasco-Torres *et al.*, 2017). Therefore, considering the cytotoxic effects of *Seidlitzia rosmarinus*, it can be suggested that this plant may weaken the male reproductive system by increasing oxidative stress and reducing the antioxidant defense function. The trend towards medical hadiths has long been common among Muslims, and in recent years, with the trend towards traditional Islamic medicine, this use is increasing. In Islamic medicine, it is said that eating *Seidlitzia rosmarinus* leads to physical weakness and corrupts the semen.

Since no study has been done on the effect of *Seidlitzia rosmarinus* on the male reproductive system, and based on the hypothesis that this plant induces cytotoxicity through disrupting antioxidant defense activity, and according to the semen’s corrosive effect of this plant in teachings Islamic medicine, the present study was conducted to consider the effect of aqueous and hydro-alcoholic extracts of *Seidlitzia rosmarinus* on gonadal and testosterone hormones, sperm count, vitality, and antioxidant enzymes level in the testis of male mice.

## MATERIALS AND METHODS

### Plant extraction

In this experimental study, 50g of *Seidlitzia rosmarinus* dry leaves were purchased from the green grocery of Ahvaz and approved by a botany specialist for aqueous extract preparation. Then, these leaves were ground and mixed with 200 ml distilled boiled water for 30 min. Then, this mixture was filtered and centrifuged at 3500 rpm for 20 min (Ahangarpour & Oroojan, 2012). To prepare a hydro-alcoholic extract, 50g of *Seidlitzia rosmarinus* leaves’ powder was dissolved in 200 ml of a mixture of water and ethanol (30-70) for 72 hr. After passing through a strainer, it was centrifuged at 3500 rpm for 20 min. Finally, the supernatant of both extracts, after drying in the incubator, was converted into powder and kept at 4°C until use (Ahangarpour *et al.*, 2017).

### Animal preparation

Twenty-four 3-month-old male NMRI mice weighing 25 to 30g were kept in a 12-hr light-dark cycle, with free access to tap water and commercial chow ad libitum. Then, the animals were divided into three groups (n=8/each): control, aqueous extract of *Seidlitzia rosmarinus*, and hydro-alcoholic extract 100 mg/kg. The extracts were administered orally for 35 days (Heidari *et al.*, 2014; Nair & Jacob, 2016).

### Hormonal and antioxidant assessment

One day after receiving the last dose of the extract, the animals were placed under deep anesthesia with a combination of ketamine-xylazine (70-10 mg/kg) (Alfasan, Netherlands). Then, blood samples were taken by cardiac puncture, and their plasma concentrations of luteinizing hormone (LH), follicle-stimulating hormone (FSH), and testosterone were measured by utilization of specific commercial kits and enzyme-linked immunosorbent assay method (Monobind, USA). Next, the left testis of the animals was isolated, homogenized, and centrifuged at 5000 rpm for 10 min using a saline phosphate buffer (Merck, Germany). Finally, the supernatant was used to measure the level of malondialdehyde, total antioxidant capacity, superoxide Dismutase, and catalase (CAT) by specific commercial kits (TebPazhouhanRazi, Iran) and quantified by colorimetry (530-540 nm).

### Testicular morphology assessment

The right testis of the animal was removed; weighted and small or large diameters were measured. Also, testicular volume was calculated according to the V = (d^2^ × π/4) L × K formula. In this formula, V is the volume of the testis, d is the small diameter and L is the large diameter of the testis, π is 3.14 and k is 0.9, a constant-coefficient (Ahangarpour *et al.*, 2014).

### Sperm analysis

The caudal part of the epididymis was separated and compressed with scissors into 6 ml of normal saline 0.9%. After stirring the mixture and homogenizing it, a drop of that was placed into each chamber of Neubauer hemocytometer (HBG. Company, Germany) and the count of sperms was done manually in the white blood cell grids under a light microscope (Labomed light microscope, USA) and data were expressed as sperm/mL. Sperm vitality was assessed by administration of eosin 1% staining (Merk Chemical Co, Germany) to separate live (unstained) and dead (red-stained) sperm. Eosin 1% was added into each chamber of the Neubauer hemocytometer and left for 30 sec, then a total of dead and live sperm were counted within 2 min (Ahangarpour *et al.*, 2014).

### Ethical consideration

The animals were treated in accordance with the principles and guidelines on animal care of Dezful University of Medical Sciences, Dezful, Iran as reviewed by an ethics committee (Code: IR.DUMS.REC.1399.027)

### Statistical analysis

Data were statistically analyzed using the SPSS statistics software v19 (international business machine IBM; USA) with one-way ANOVA and post hoc LSD tests. The results were presented as Mean±SEM (standard error of means) and *p*≤0.05 is considered significant in all experiments.

## RESULTS

### Testicular weight and morphology

Testicular morphology assessment indicated no significant differences in testis weight, length, width, and volume in all experiment groups (Table 1).

**Table 1:** The role of aqueous and hydro-alcoholic extracts of *Seidlitzia rosmarinus* on weight, morphology, lipid peroxidation, and antioxidant enzymes of testis.

Groups	Testis weight (mg)	Testis length (mm)	Testis width (mm)	Testis volume (mm^3^)	MDA (µM)	TAC (µM)	SOD (U/mL)	CAT (U/mL)
Control	87.75±6.68	7.25±0.25	4.25±0.25	94.67±15.54	0.63±0.04	309.99±12.78	95.61±9.61	226.26±9.53
Aqueous extract	90.66±8.86	7.00±0.40	4.50±0.28	102.97±17.56	0.91±0.08^*^	241.10±11.15^**^	92.67±8.17	91.19±13.91^***^
Hydro- alcoholic extract	99.50±10.91	6.75±0.25	4.50±0.25	96.57±12.45	0.95±0.07^*^	229.25±14.54^**^	95.30±7.63	175.77±6.55^*^

Data are presented as mean ± standard error of means; n=8.

* *p*<0.05,

** *p*<0.01 and

*** *p*<0.001 compared with the Control group. MDA: Malondialdehyde; TAC: Total antioxidant capacity; SOD: Superoxide dismutase; CAT: Catalase.

### Lipid peroxidation and antioxidant alterations

Present results showed a significant increase of malondialdehyde level in aqueous and hydro-alcoholic extracts groups compared to the control group (*p*=0.04). The testis’ levels of total antioxidant capacity decreased in aqueous, and hydro-alcoholic (*p*=0.008) extracts administered in animals when compared with the control group. Moreover, consumption of aqueous (*p*<0.001) and hydro-alcoholic (*p*=0.03) extracts of *Seidlitzia rosmarinus* decreased the level of testicular CAT in comparison with that of the control group. Finally, there were no significant changes in the testis superoxide dismutase levels between experiment groups (Table 1).

### Changes in male sex hormones in control and treated groups

**Figure 1:**
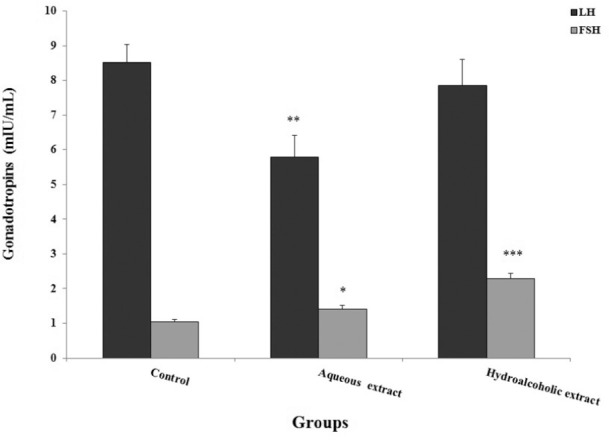
Effects of *Seidlitziarosmarinus* extracts on plasma level of gonadotropin hormones. The data are expressed as the mean ± standard error of means. (n=8). *p<0.05, **p<0.01 and ***p<0.001, significantly different from the control group. LH: Luteinizing hormone; FSH: Follicle-stimulating hormone.

**Figure 2:**
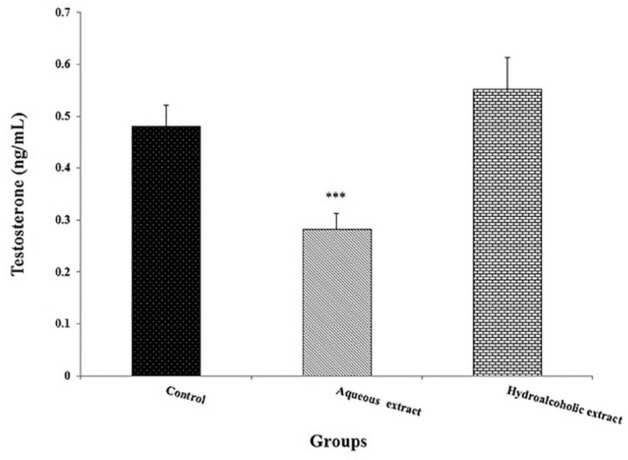
Effects of *Seidlitzia rosmarinus* extracts on plasma levels of testosterone. Data are expressed as the mean ± standard error of means (n=8). ***p<0.001, significantly different from the control group.

The plasma level of LH decreased in the aqueous extracts-administrated group compared with the control animals (*p*=0.009). The level of FSH increased with the aqueous (*p*=0.03) and hydro-alcoholic (*p*<0.001) extracts administered in mice compared to the controls (Figure 1). Also, testosterone measurement plasma level showed a significant decreased in the aqueous extracts of *Seidlitzia rosmarinus-*treated animals *versus* the control group (*p*<0.001; Figure 2).

### Effect of *Seidlitzia rosmarinus* extracts on sperm count and vitality

**Figure 3:**
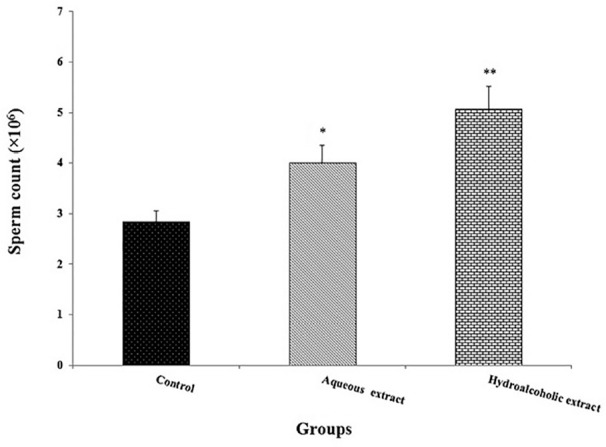
Effects of *Seidlitzia rosmarinus* extracts on sperm count. Data are expressed as the mean ± standard error of means (n=8). *p<0.05 and **p<0.01 significantly different from the control group.

**Figure 4:**
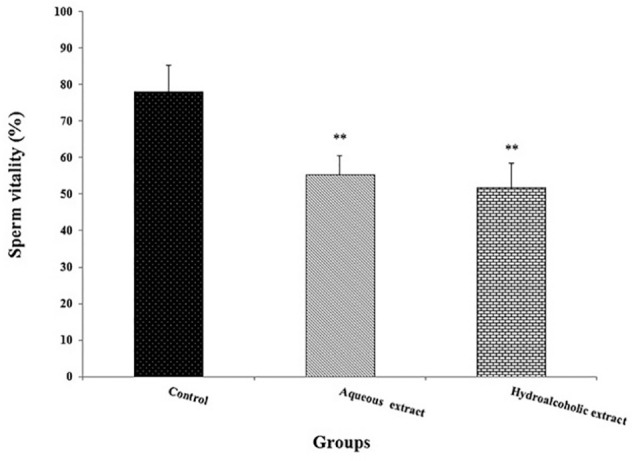
Effects of *Seidlitzia rosmarinus* extracts on sperm vitality. Data are expressed as the mean ± standard error of means (n=8). **p<0.01 significantly different from the control group.

However, the number of sperms was increased in aqueous (*p*=0.04) and hydro-alcoholic (*p*=0.009; Figure 3) extracts groups the percentage of sperm vitality was decreased (*p*=0.008) in those groups when compared to the control group (Figure 4).

## DISCUSSION

The present study results showed that the aqueous extract of *Seidlitzia rosmarinus* decreased plasma level of LH and testosterone while both extracts increase the plasma level of FSH that was characterized by an increase in sperm count of the mice treated with aqueous and hydro-alcoholic extracts of *Seidlitzia rosmarinus*. Gonadotropins, such as FSH, in addition to their importance for ovary function they are necessary for normal testis activities such as spermatogenesis (Glage *et al.*, 2013). For example, O’Shaughnessy *et al*. (2010) demonstrated that FSH stimulates the spermatogenesis process via an increase in spermatogonial number and entry into meiosis. FSH is a pituitary secreted hormone with structural and metabolic effects on the spermatogenesis process by increasing Sertoli cells count and its membrane-bound receptor for this hormone. These alterations lead to the development of spermatogonia into mature spermatids and increase sperm count (Oduwole *et al.*, 2018). So, concomitant with the present results it could be suggested that *Seidlitzia rosmarinus* increases the number of sperm in treated mice by enhancing the plasma level of FSH. Another variable was sperm vitality in the present study, that revealed a significant decrease in *Seidlitzia rosmarinus-*administered mice. However, this plant increased sperm count but led to increased induction of sperm death in these animals. Therefore, this plant has a spermicidal action consistent with the statements made in Islamic medicine regarding the corrosive function of this plant on the semen.

Lipid peroxidation and antioxidant status assessment of this study indicated that administration of aqueous and hydro-alcoholic extracts of *Seidlitzia rosmarinus* increased lipid peroxidation and decreased total antioxidant capacity as well as CAT. The spermatozoal membrane has a lot of polyunsaturated fatty acids (PUFAs) that cause the increase of sperm susceptibility to oxygen-induced damage mediated by lipid peroxidation, and this feature leads to reduce sperm viability (Van Tran *et al.*, 2017). Catalase plays a critical role in sperm and controls the oxidative stress in cells, especially resulting from H_2_O_2_. This antioxidant enzyme converts H_2_O_2_ as a free radical to O_2_ and H_2_O, which removes it (Rubio-Riquelme *et al.*, 2020). Oxidative stress induced by H_2_O_2_ disrupts sperm motility and vitality and destroys all the sperm functions that lead to fertilization such as sperm-oocyte fusion. This finding indicated that increased levels of H_2_O_2_ in spermatozoa might be an effective way of contraception and male infertility (Bansal & Bilaspuri, 2010). So, according to the present results, it could be suggested that *Seidlitzia rosmarinus* reduced sperm vitality by increasing lipid peroxidation and H_2_O_2_ in the testis. Moreover, according to the previous study, LH and testosterone are essential for sperm vitality (Orieke *et al.*, 2019). Hence, it can be suggested that one of the causes of decreased sperm viability in the *Seidlitzia rosmarinus* aqueous extract-treated group, in addition to increasing oxidative stress, it is a decrease in the levels of LH and testosterone.

## CONCLUSION

In conclusion, the present study demonstrated that *Seidlitzia rosmarinus* has an adverse effect on male reproductive hormones and sperm viability via increased lipid peroxidation and disrupting antioxidants’ defense system balance. However, both extracts of this plant increased sperm count, but this effect occurred in dead sperm instead of living sperm. Therefore, according to Islamic medicine, it could be suggested that *Seidlitzia rosmarinus* can induce corruption in sperm and eventually semen.
